# Spectrophotometric methods for determination of zolpidem tartrate in tablet formulation

**DOI:** 10.4103/0975-7406.72142

**Published:** 2010

**Authors:** Rajiv Chomwal, Amit Kumar, Anju Goyal

**Affiliations:** Department of Pharmaceutical Chemistry, Swami Keshvanand Institute of Pharmacy, Bikaner 334001, India; 1 Department of Pharmaceutical Chemistry, BNPG Girls College of pharmacy, Udaipur, Rajasthan, India

**Keywords:** Spectrophotometric, quantitative estimation, zolpidem tartrate

## Abstract

**Aim:**

The study aims to develop simple, sensitive, rapid, accurate and precise spectrophotometric method for estimation of Zolpidem tartrate in tablet dosage forms.

**Materials and Methods:**

For method I, II, III and IV in a series of 10 ml volumetric flask, aliquots of standard drug solution (100 *µ*g/ml) in 0.1N HCl were transferred and diluted with same so as to give several dilutions in concentration range of 5-30 *µ*g/ml, 5-30 *µ*g/ml, 10-50 *µ*g/ml and 5-40 *µ*g/ml respectively of zolpidem tartrate. To 5 ml of each dilution taken in a separating funnel, (5 ml of bromo phenol red, bromo cresol purple, bromo cresol green and bromo phenol blue for method I, II, III and IV respectively) reagent and 5 ml of chloroform was added. Reaction mixture was shaken gently for 5 min and allowed to stand so as to separate aqueous and chloroform layer. Absorbance maxima measured at 407 nm, 417 nm, 412 nm and 415 nm for method I, II, III and IV respectively.

**Results:**

The recovery studies were found close to 100 % that indicates accuracy and precision of the proposed methods. The statistical analysis was carried out and results of which were found satisfactory. Standard deviation values were found low that indicated reproducibility of the proposed methods.

**Conclusion:**

Based on results the developed methods could be used for routine estimation of zolpidem tartrate from tablet formulations.

Zolpidem tartrate, chemically bis [N, N dimethyl-2[6-methyl-2-(4-methyl phenyl)] imidazo[1, 2-α] pyridine-3-yl]acetamide](2R,3R)-2,3dihydroxybutane dioate, is a hypnotic agent.[1] It produces agonistic effect on GABA_A_ receptors and it is used in the treatment of insomnia. It is official in BP^1^ and Merck Index^2^ which describes liquid chromatographic method for its quantitation.[1,2] Literature survey reveals that four HPLC methods,[3–6] one potentiometric method[7] have been developed for the estimation of zolpidem tartrate in human serum and tablet formulation. The objective of the present investigation has been to develop simple, accurate and economical visible spectrophotometric methods for quantitation of zolpidem tartrate in tablet formulation.

## Materials and Methods

Shimadzu UV 1700, UV/Vis double beam spectrophotometer with spectral band width of 1 nm, wavelength accuracy of ± 0.3 nm and 1.0 cm matched quartz cells was used for analytical method development. All the chemicals and reagents used were of analytical grade. Bromo phenol red reagent (Merck Germany), bromo cresol purple, bromo cresol green, bromo phenol blue (Loba Chemie, Mumbai) reagents were prepared in double distilled water. All the reagents were extracted several times with chloroform so as to remove chloroform soluble impurities. Tablet formulations of zolpidem tartrate [Nitrest (Sun Pharmaceutical Industries, Dadra), Zolfresh (Acme formulation Pvt. Ltd., Solan)] were procured from local pharmacy. Standard solution of zolpidem tartrate was prepared by dissolving 10 mg in 100 ml of 0.1N HCl solution to give stock solution of concentration 100 *µ*g/ml of drug.

### Procedure for preparation of calibration curve

**Figure 1:**
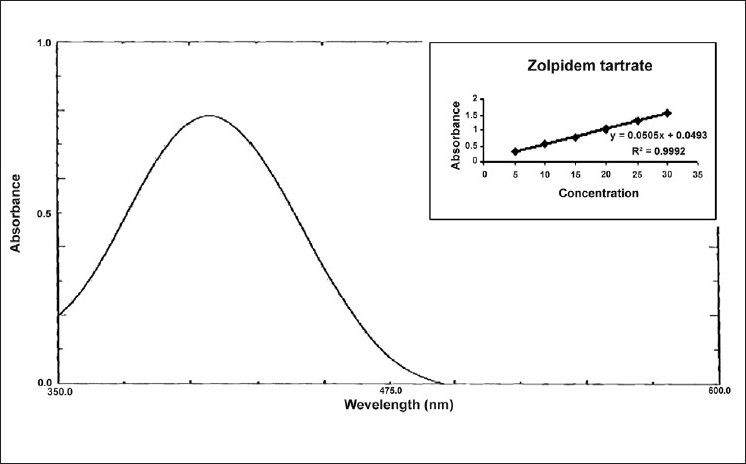
UV spectrum of zolpidem tartrate with bromo phenol red reagent

For method I, in a series of 10 ml volumetric flask, aliquots of standard drug solution

(100 *µ*g/ml) in 0.1N HCl solution were transferred and diluted with same so as to give several dilutions in concentration range of 5-30 *µ*g/ml of zolpidem tartrate. To 5 ml of each dilution taken in a separating funnel, 5 ml of bromo phenol red (0.2 % w/v) reagent and 5 ml of chloroform was added. Reaction mixture was shaken gently for 5 min and allowed to stand so as to separate aqueous and chloroform layer. The chloroform layer was separated out and an absorbance maximum was measured at 407.0 nm [Figure 1] against a reagent blank. Calibration curve was plotted [Figure 1] between concentration of zolpidem tartrate and measured absorbance. Spectral characteristics of zolpidem tartrate are given in Table 1.

**Figure 2:**
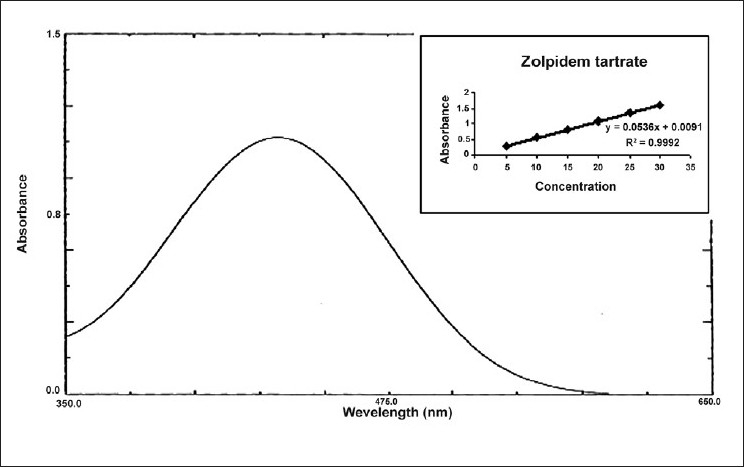
UV spectrum of zolpidem tartrate with bromo cresol purple reagent

For method II, in a series of 10 ml volumetric flask, aliquots of standard drug solution (100 *µ*g /ml) in 0.1NHCl solution were transferred and diluted with same so as to give several dilutions in concentration range of 5-30 *µ*g/ml of zolpidem tartrate. To 5 ml of each dilution taken in a separating funnel, 5 ml of bromo cresol purple reagent (0.5 % w/v) and 5 ml of chloroform was added. Reaction mixture was shaken gently for 5 min and allowed to stand so as to separate aqueous and chloroform layer. The chloroform layer was separated out and an absorbance maximum was measured at 417.0 nm [Figure 2] against a reagent blank. Calibration curve was plotted [Figure 2] between concentration of zolpidem tartrate and measured absorbance. Spectral characteristics of zolpidem tartrate are given in Table 1.

**Figure 3:**
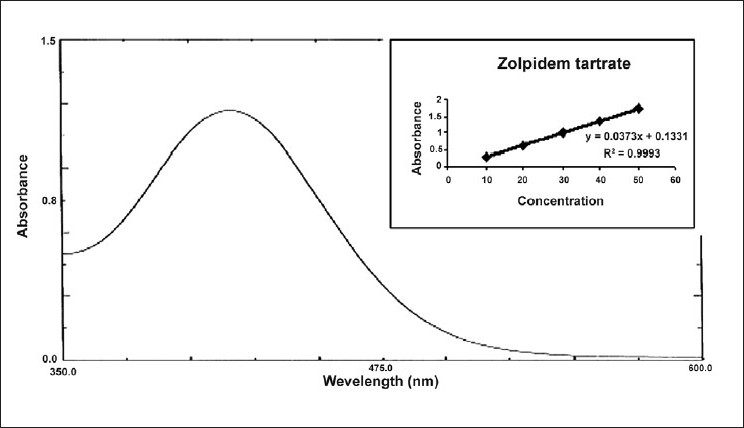
UV spectrum of zolpidem tartrate with bromo cresol green reagent

For method III, in a series of 10 ml volumetric flask, aliquots of standard drug solution (100 *µ*g/ ml) in 0.1N HCl solution were transferred and diluted with same so as to give several dilutions in concentration range of 10-50 *µ*g/ml of zolpidem tartrate. To 5 ml of each dilution taken in a separating funnel, 5 ml of bromo cresol green reagent (0.2 % w/v) and 5 ml of chloroform was added. Reaction mixture was shaken gently for 5 min and allowed to stand so as to separate aqueous and chloroform layer. The chloroform layer was separated out and an absorbance maximum was measured at 412.0 nm [Figure 3] against a reagent blank. Calibration curve was plotted [Figure 3] between concentration of zolpidem tartrate and measured absorbance. Spectral characteristics of zolpidem tartrate are given in Table 1.

**Table 1:** Spectral characteristics of zolpidem tartrate

Parameters	Method I	Method II	Method III	Method IV
λ_max_	407.0 nm	417.0 nm	412.0 nm	415.0 nm
Beer’s law limit (*µ*g/ml)	5-30 *µ*g/ml	5-30 *µ*g/ml	10-50 *µ*g/ml	5-40 *µ*g/ml
Regression equation* A +bc	y = 0.0505 + 0.0493c	y = 0.0536 + 0.0091c	y = 0.0373 − 0.1331c	y = 0.0452 + 0.1914c
Slope (b)	0.0493	0.0091	0.1331	0.1914
Intercept (a)	0.0505	0.0536	0.0373	0.0452
Correlation coefficient (r^2^)	0.9992	0.9992	0.9993	0.9992

y = a + bc, where c is the concentration in *µ*g/ml, y is the absorbance unit of five replicate samples and b is the slope of line equation.

**Figure 4:**
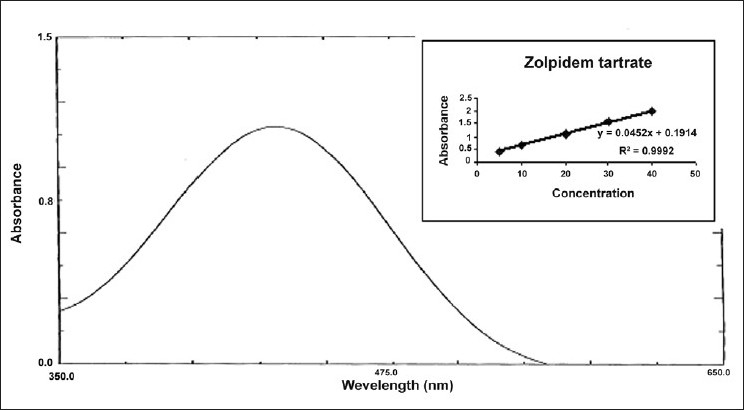
UV spectrum of zolpidem tartrate with bromo phenol blue reagent

For method IV, in a series of 10 ml volumetric flask, aliquots of standard drug solution (100*µ*g/ml) in 0.1N HCl solution were transferred and diluted with same so as to give several dilutions in concentration range of 5-40 *µ*g/ml of zolpidem tartrate. To 5 ml of each dilution taken in a separating funnel, 5 ml of bromo phenol blue reagent (0.2 % w/v) and 5 ml of chloroform was added. Reaction mixture was shaken gently for 5 min and allowed to stand so as to separate aqueous and chloroform layer. The chloroform layer was separated out and an absorbance maximum was measured at 412.0 nm [Figure 4] against a reagent blank. Calibration curve was plotted [Figure 4] between concentration of zolpidem tartrate and measured absorbance. Spectral characteristics of zolpidem tartrate are given in Table 1.

### Procedure for analysis of tablet formulation

For analysis of tablet formulation, 20 tablets (5 mg and 10 mg) of zolpidem tartrate were accurately weighed and finely powdered. An accurately weighed powdered sample 10 mg of zolpidem tartrate was taken in a 100 ml volumetric flask containing 40 ml of 0.1N HCl solution, sonicated for 10 min. The resultant was filtered through Whatman filter paper no. 41 into another 100 ml volumetric flask. The filter paper was washed several times with 0.1N HCl solution. The washings were added to the filtrate and final volume was made up to the mark with 0.1N HCl solution.

For method I, 1 ml of filtrate of the sample solution was diluted to 10 ml with 0.1N HCl solution. These were treated as per the procedure for preparation of calibration curve and amount of the drug present in sample was computed from respective calibration curve.

For method II, 1 ml of filtrate of the sample solution was diluted to 10 ml with 0.1N HCl solution. These were treated as per the procedure for preparation of calibration curve and amount of the drug present in sample was computed from respective calibration curve. For method III, 3 ml of filtrate of the sample solution was diluted to 10 ml with 0.1N HCl solution. These were treated as per the procedure for preparation of calibration curve and amount of the drug present in sample was computed from respective calibration curve. For method IV, 1 ml of filtrate of the sample solution was diluted to 10 ml with 0.1N HCl solution. These were treated as per the procedure for preparation of calibration curve and amount of the drug present in sample was computed from respective calibration curve.

The procedure of analysis from tablet formulations for all the methods was repeated five times with two different tablet formulations and results are reported in Table 2.

**Table 2:** Results of analysis and recovery studies of commercial formulations of zolpidem tartrate

Method	Formulation	Label claim (mg/tab)	% Label claim*	% Recovery**	SD
I	Nitrest-5	5	100.36	99.53	0.450
	Zolfresh-10	10	99.62	99.38	0.703
II	Nitrest-5	5	99.40	98.91	0.616
	Zolfresh-10	10	99.57	99.40	0.740
III	Nitrest-5	5	99.56	99.59	0.676
	Zolfresh-10	10	99.61	99.19	0.550
IV	Nitrest-5	5	99.77	99.01	0.590
	Zolfresh-10	10	99.68	99.39s	0.530

* Average of five determinations.

** Average of determinations at three different concentration levels.

SD- Standard deviation

### Recovery studies

Recovery studies were carried out for both the developed methods by addition of known amount of standard drug solution of zolpidem tartrate to pre-analyzed tablet sample solution at three different concentration levels. The resulting solutions were analyzed by proposed methods. The recovery was in the range of 99-100 % for method I, 98-100 % for method II, 99-100 % for method III and 99-100% for method IV. The results of recovery studies are reported in Table 2.

## Result and Discussion

The development of a new dosage form involves a number of stages and the analytical methods that are specific, accurate and precise, plays a vital role in many of the essential features required for an identical analytical system, have been adopted in a wide range of pharmaceutical analysis. Taking into account the above, four accurate, simple, precise, economical and rapid visible spectrophotometric assay methods were developed for the quantitative estimation of zolpidem tartrate in tablet dosage forms.

The proposed methods obeyed Beer’s law in the concentration range of 5-30 *µ*g/ml with bromo phenol red 5-30 *µ*g/ml with bromo cresol purple, 10-50 *µ*g/ml with bromo cresol green and 5-40 *µ*g/ml with bromo phenol blue reagent. The recovery studies were carried out by standard addition method and were found close to 100 % that indicates accuracy and precision of the proposed methods. The statistical analysis was carried out and results of which were found satisfactory. Standard deviation values were found to be low that indicated reproducibility of the proposed methods. It was observed that excipients did not interfere with the determination of zolpidem tartrate. Hence, these developed methods could be used for routine estimation of zolpidem tartrate from tablet formulations.

## Conclusion

The developed methods were found to be precise and accurate. The methods can be used for the routine spectrophotometric analysis of zolpidem tartrate in pharmaceutical preparations. Despite the low concentration of zolpidem tartrate, these methods were successfully used to estimate the amount of zolpidem tartrate present in the tablet without the addition of internal standard or prior separation. Moreover, the developed methods have the advantages of simplicity, convenience and quantification of zolpidem tartrate for the assay of their dosage form.
